# Effects of 25 mg oxazepam on emotional mimicry and empathy for pain: a randomized controlled experiment

**DOI:** 10.1098/rsos.160607

**Published:** 2017-03-08

**Authors:** Gustav Nilsonne, Sandra Tamm, Armita Golkar, Karolina Sörman, Katarina Howner, Marianne Kristiansson, Andreas Olsson, Martin Ingvar, Predrag Petrovic

**Affiliations:** 1 Department of Clinical Neuroscience, Karolinska Institutet, Stockholm, Sweden; 2 Stress Research Institute, Stockholm University, Stockholm, Sweden; 3 Department of Clinical Psychology, University of Amsterdam, Amsterdam, The Netherlands; 4 Department of Neuroradiology, Karolinska University Hospital, Stockholm Sweden

**Keywords:** emotional mimicry, emotional contagion, empathy, benzodiazepines, psychopathic traits

## Abstract

Emotional mimicry and empathy are mechanisms underlying social interaction. Benzodiazepines have been proposed to inhibit empathy and promote antisocial behaviour. First, we aimed to investigate the effects of oxazepam on emotional mimicry and empathy for pain, and second, we aimed to investigate the association of personality traits to emotional mimicry and empathy. Participants (*n*=76) were randomized to 25 mg oxazepam or placebo. Emotional mimicry was examined using video clips with emotional expressions. Empathy was investigated by pain stimulating the participant and a confederate. We recorded self-rated experience, activity in major zygomatic and superciliary corrugator muscles, skin conductance, and heart rate. In the mimicry experiment, oxazepam inhibited corrugator activity. In the empathy experiment, oxazepam caused increased self-rated unpleasantness and skin conductance. However, oxazepam specifically inhibited neither emotional mimicry nor empathy for pain. Responses in both experiments were associated with self-rated empathic, psychopathic and alexithymic traits. The present results do not support a specific effect of 25 mg oxazepam on emotional mimicry or empathy.

## 1. Introduction

Facial emotional signals are important for human social interaction [1]. Lipps proposed in 1907 that observation of emotional expression leads to mimicry and a convergence of subjective emotional states [2]. Hatfield *et al.* [3] have defined emotional contagion as ‘the tendency to automatically mimic and synchronize expressions, vocalizations, postures, and movements with those of another person’s and, consequently, to converge emotionally’. Thus, emotional contagion is a mirroring of another’s internal emotional state, of which emotional mimicry forms a part.

Several researchers have theorized that emotional mimicry is a basic mechanism for sharing of emotions, on which more complex forms of empathy are based [4–6]. Putative evolutionary fitness advantages include improved coordination and sharing of important information in a social group by aligning emotional/motivational states [7], and improved attachment, facilitating prosocial behaviour [8]. The former of these two selection mechanisms is supported by findings that emotional mimicry has been shown to correlate to accurate decoding of emotional expressions, although this effect remains controversial [9]. The latter putative selection mechanism is supported by findings that mimicry is greater to in-group members and that it increases liking for in-group members more than it does to out-group members [8], and that facial emotional mimicry correlates to prosocial behaviour [10], as does empathy for pain [11].

Empathy for pain has been investigated using functional brain imaging for more than a decade [12]. A consistent finding is that observation of pain in others is associated with activation in the anterior insula and anterior midcingulate cortex [12,13]. This result is consistent with simulation theory, according to which others’ emotional states are understood through a representation in brain networks overlapping with those that represent one’s own internal states [14–16]. The meaning of these overlapping activations, and the extent to which they provide evidence for shared representations, is an area of active debate [12,17,18]. Psychometric research has defined facets of empathy using factor analyses of self-rated data. One influential categorization differentiates between empathic concern, personal distress, perspective taking and fantasy [19,20]. A notable finding in early brain imaging studies of empathy for pain was that activity in the insula correlated to self-rated empathic concern [12]. This finding has, however, not replicated well [13]. In studies of empathy for pain, behavioural outcomes are nonetheless expected to be predicted by self-rated empathic concern as well as personal distress. Regardless of precise mechanisms, empathic representation of others’ emotions has been proposed as a major contributor to prosocial behaviour [21,22], and specifically a mechanism to prevent violent behaviour against the person with whom empathy is felt.

In forensic psychiatric case series [23–25], Dåderman *et al.* have reported instrumental use of benzodiazepines, particularly flunitrazepam, to facilitate violent criminal behaviour, raising concerns that these drugs may inhibit empathic responses. These findings are consistent with earlier reports of paradoxical reactions with increased agitation and aggressiveness following benzodiazepine use [26–29]. Recent epidemiological data offer further evidence: a case–control study from Finland found higher rates of benzodiazepine prescriptions for persons convicted of homicide compared with other offenders [30], and a retrospective analysis of toxicology reports from persons convicted of homicide in Sweden found a prevalence of benzodiazepine use of 19% [31]. These studies are limited by their observational nature; in particular, it is hard to rule out the possibility that participants who received benzodiazepines had different signs and symptoms of psychopathology than those who did not (confounding by indication). In male rats, midazolam, triazolam and flunitrazepam have been found to increase aggressive behaviour [32,33].

Benzodiazepines act by potentiating GABA_A_ receptors, which are pentameric ligand-gated ion channels composed of *α*, *β* and *γ* subunits. The GABA binding site is located at the interface of *α* and *β* subunits, and the allosteric benzodiazepine binding site is located homologously at the interface between *α* and *γ* subunits. In humans, six types of the *α* subunit have been discovered, which are variably expressed in different brain areas and to which different benzodiazepines bind with varying affinity. Anxiolytic effects of benzodiazepines are thought to be mediated mainly by *α*-2 subunit containing GABA_A_ receptors [34], which are strongly expressed in the amygdala [35]. Sedative and anticonvulsant effects are thought to be mediated mainly by *α*-1 subunit containing GABA_A_ receptors, which are expressed widely in the cerebral cortex [34–37]. Effects of the benzodiazepines diazepam and lorazepam on recognition of emotional expressions have been previously investigated [38–42]. Impairment of emotion recognition was found in studies using 15 mg diazepam [38–40], but not in studies using 5 mg diazepam [42] nor 2 mg lorazepam [41]. Effects of benzodiazepines on emotional mimicry and empathy for pain have not, to the best of our knowledge, been investigated before. An important consideration in behavioural experiments using benzodiazepines is that the dose should be sufficiently high to permit investigation of effects of interest, while not so high as to sedate the participants. Equipotent dosages for benzodiazepines have been determined mainly for clinical purposes, and a relatively low dose of 25 mg oxazepam is comparable to a dose of 15 mg diazepam [43].

### 1.1. Aims

Because benzodiazepines have been reportedly used to facilitate aggressive and violent behaviour, we hypothesized that benzodiazepines would inhibit empathic responding. Therefore, we aimed to investigate the effect of 25 mg oxazepam, a commonly prescribed benzodiazepine, on emotional mimicry and empathic responding, using subjective and physiological measures.

## 2. Material and methods

### 2.1. Study design

The study was a double-blind randomized controlled experiment performed in two waves. We investigated the effect of oxazepam on three different emotional processes: empathy for pain, emotional mimicry and emotion regulation by cognitive reappraisal. This paper describes the experiments on mimicry and empathy for pain. The reappraisal experiment will be reported elsewhere. In wave 2, timing of stimulus presentation was revised and heart rate was added as an outcome measure (see below).

### 2.2. Participants

Healthy male volunteers were recruited by advertisement on university campuses in Stockholm, Sweden, and using a website (www.studentkaninen.se). Participants were required to be right-handed, male, 18–45 years of age, to have no history of neurological or psychiatric disease including substance abuse, to speak and understand Swedish fluently and not to be habitual consumers of nicotine. Furthermore, students of psychology, behavioural sciences and medicine (past the third semester) were not included, because we thought they might be more likely to try to uncover the role of the confederate, and because training in medicine probably causes a more detached attitude towards images of injured and sick people, which were used in the reappraisal experiment. We recruited only male participants, because the earlier work on criminal offenders as well as experimental animals was restricted to males (see Introduction), and a study investigating sex differences in brain mechanisms showed that males have a greater capacity for downregulating empathic responses [21]. We aimed for a sample size of *n*=40 for each wave, with 20 participants in each of the two treatment groups, based on pragmatic considerations. Participants were paid 500 SEK (approx. 50 Euro or 60 USD), subject to tax.

### 2.3. Procedures

#### 2.3.1. Screening, instructions and intervention

On arrival, participants were allowed to acquaint themselves for a few minutes with a confederate who was introduced as another experimental participant, but who was in reality a fellow investigator (S.T.), through a short scripted interaction.

Participants completed a brief medical screening form to verify that they fulfilled inclusion criteria. They were given written and oral information about the experiment and gave written informed consent. Next, they were given either a tablet of 25 mg oxazepam or a placebo pill, for which we used non-prescription vitamin D3 supplement pills of similar size and shape. Tablets were in pre-prepared sealed envelopes, and both the investigators and the participants were blind to the treatment condition. We chose to use oxazepam because it has a favourable side-effect profile and relatively weak sedative effects compared with other benzodiazepines. We used a dose of 25 mg hoping that it would not have so strong subjective effects as to break blinding. Following oral administration, oxazepam reaches its maximal plasma concentration after about 2 h, and maximum brain concentrations about half an hour after that [44–46]. Elimination occurs through glucuronidation yielding no active metabolites, with a half-life of 5–15 h [44,45]. Participants were instructed not to drive until the next day, in order to reduce risks from sedative effects in traffic.

Immediately after administration of drug or placebo, participants completed a reaction time task, titration of pain thresholds and several rating scales. These baseline measures were recorded immediately after drug administration rather than before, in order to use time efficiently, based on the assumption that effects of oxazepam would only appear later (at least 20 min after ingestion). Approximately 45–60 min after drug administration, participants underwent the mimicry for pain experiment and the empathy for pain experiment. Later, they also underwent the experiment on emotional reappraisal. At debriefing after the experiment, the role of the confederate was revealed, participants were asked to rate the confederate’s likability, and we asked an open-ended question about the participants’ experience.

Participants were block-randomized in groups of four to oxazepam or placebo, and to two different orders of stimulus presentation, meant to be counterbalanced between treatment groups. However, owing to an error in the randomization procedure in wave 1, stimulus presentation order was instead conflated with treatment groups. We judged this to be a very minor problem for the mimicry and empathy experiments, but in the reappraisal experiment, it caused stimulus images to not be balanced with respect to reappraisal instructions.

#### 2.3.2. Reaction time test

The purpose of the reaction time test was to measure vigilance, in order to gain an independent measure of the effect of oxazepam. The test was administered on a desktop personal computer using the Presentation software (Neurobehavioural Systems, Berkeley, CA). At intervals randomized between 2 and 10 s, a 200×200 pixel white square was shown at a random location on the screen for 1 s. Participants were instructed to press the space bar as fast as possible when the square appeared. There were 40 events, for an average length of 4 min for the whole test. Responses slower than 1 s were considered lapses and responses faster than 100 ms would have been considered false starts, had there been any. The outcome of interest was response time, as the test was too short to be sensitive for lapses. Response times were inverse-transformed to better approximate a normal distribution, which is a well-established practice for vigilance tests [47]. Stimulus presentation code is available at [48].

#### 2.3.3. Experimental paradigm for emotional mimicry

To induce emotional mimicry, we constructed two sets of video stimuli. In the first set, we recorded professional actors. However, having used the first set in wave 1, it was felt that their emotional expressions bore marks of professional training, and we found that one of the actors was sometimes recognized by the participants. Therefore, we constructed a second stimulus set for wave 2, where we recorded young non-actors instead. One identity from the first stimulus set was retained in the second set. Models were recorded against a white background while wearing identical grey T-shirts. They were filmed while moving from a neutral facial expression to an angry or a happy expression, or maintaining the neutral expression. Film clips were 6 (wave 1) or 4 s (wave 2) long, with the change from neutral expression beginning 2 s into the clip. All stimuli are available at [48].

**Figure 1.**
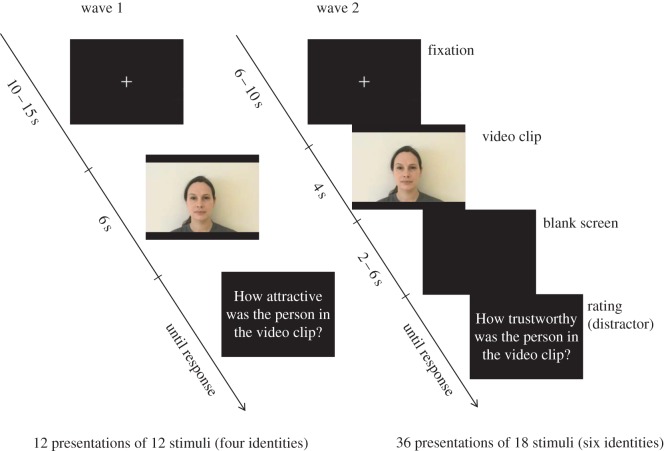
Stimulus sequence for the emotional mimicry experiment. In wave 2, a jitter was introduced between the video clip and the rating, so the experiment could later be converted into an fMRI experiment without conflation of effects of the video clip and of the rating in statistical modelling. Rating questions were presented in Swedish, but are shown here translated to English.

Video clips were shown using the Presentation software (Neurobehavioral systems, Inc., Berkeley, CA) on a computer screen. Figure 1 shows timing details. A distractor question was asked of the participants after each video clip, in order to decrease the risk that they would realize their facial expressions were recorded. In wave 1, we asked the participants to rate from 0 to 100 how attractive the model was. Because the models were of both sexes, and the participants were not screened based on sexual orientation, we changed the question in wave 2 to a rating of trustworthiness instead, using the same scale of 0–100. In wave 2, changes were also made to improve timing by shortening the stimulus presentation time and adding a jittered pause between the video clip and the rating. The purpose of jittering was to enable direct translation of the paradigm into a functional magnetic resonance imaging setting for future studies. Also, the number of stimulus presentations was increased from wave 1 to wave 2 by adding two more model identities (figure 1). Every model was shown once with each of the three emotional expressions. Stimulus presentation code is available at [48].

#### 2.3.4. Experimental paradigm for empathy for pain

The experiment on empathy for pain is adapted from Singer *et al.* [12]. Participants were seated in front of a table with a computer monitor, and asked to lay their right arm, on which we had placed the stimulus electrode, in the table. The confederate was seated next to the participant with her arm on the table. A screen was placed on the floor between the participant and the confererate, so they could see each other’s extended arms only.

**Figure 2.**
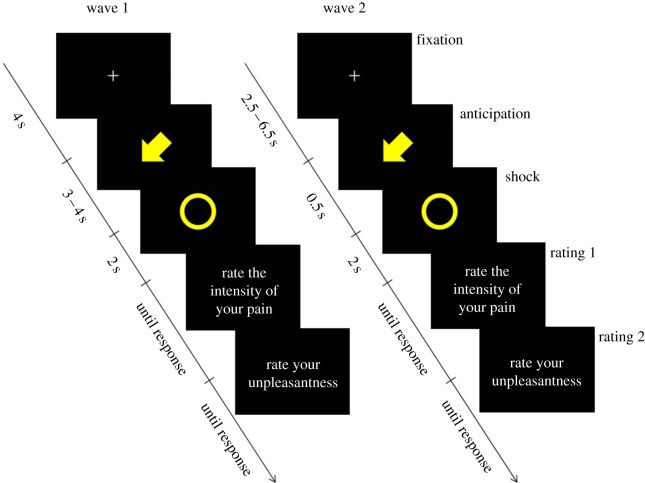
Stimulus sequence for the empathy for pain experiment. In wave 2, timing was optimized to reduce uncertainty about the contribution of anticipation to observed responses. Shown here are stimuli for a low-intensity shock to the participant. In half of the trials, the fixation cross was followed instead by a rest event of 5.5 s. In wave 2, fixation crosses after rest events were jittered not between 2.5 and 6.5 s but between 1 and 5 s, to save time. Rating questions were presented in Swedish, but are shown here translated to English.

There were a total of 40 shock events and 40 ‘null’ events. For every shock event, a cue was shown on the computer monitor, in the form of an arrow pointing at either the participant or the confederate and with different colours for the participant and the confederate. Low-intensity shocks were cued by a solid-colour arrow, and high-intensity shocks by a striped arrow. At the same time as the shock, a circle was shown on the screen, colour-coded in the same manner as the arrows. Timing is described in figure 2. In wave 2, we shortened the anticipation time in order to better be able to study the effects of the shock itself, rather than effects owing to prolonged anticipation (figure 2). Stimulus presentation code and materials are available at [48].

#### 2.3.5. Pain stimulation

We used a custom-built concentric stimulation electrode consisting of a non-ferromagnetic conducting element of approximately 4 mm ø, insulated by a plastic ring of about 3 mm, surrounded by another conducting element of approx. 1 mm, insulated on the outside by another layer of plastic. We placed the electrode on the volar forearm in order to avoid muscle contractions. Spectra 360^®^ contact gel (GEL104, Biopac Systems, Inc., Goleta, CA) was used. The electrode was connected to a Biopac recording system with an STM200 stimulation unit (Biopac Systems, Inc.). Shocks lasted for 200 ms. In order to achieve comparable pain intensities, pain thresholds were titrated individually for each participant using a visual analogue scale (VAS) from 0 to 100. For each participant, we identified VAS 10 (perceptible but not painful) and VAS 80 (as painful as they considered to be bearable for the experiment). Titration was repeated at the end of the experiment to verify that pain perception as such had not been inhibited by oxazepam.

#### 2.3.6. Skin conductance

Skin conductance responses were measured using two 6 mm ø Ag/AgCl finger electrodes (TSD203, Biopac Systems, Inc.) with isotonic 0.05 M NaCl electrode paste (GEL101, Biopac Systems, Inc.), connected to a GSR100C amplifier (Biopac Systems, Inc.) with the following acquisitions settings: $5\;\text{μ℧}\;V^{-1}$, 1 Hz low-pass filter and direct current. To remove non-physiological noise, data were further filtered in the Acqknowledge software using a low-pass filter with a 1 Hz cutoff and 4000 coefficients and converted from direct to alternating current using a 0.05 Hz high-pass filter. Responses were identified manually after each stimulus by inspection of the curve in the interval from cue onset to 2 s after shock onset. It was not possible to differentiate responses to the cue and responses to the shock, and the greatest response in the interval was recorded. A response was defined as a wave starting from a slope of 0, unless the baseline was trending upwards, in which case the point with the lowest slope (derivative) was used as baseline. Amplitude was defined as the height of the peak, which was allowed to be anywhere within 6 s from onset, in microsiemens. If no peak appeared within 6 s, the response was excluded from analysis. Data were square root transformed before statistical analysis, in order to better approximate a normal distribution.

#### 2.3.7. Electromyography

Electromyography (EMG) was measured over the superciliary corrugator muscles following established guidelines [49]. In the experiment on empathy for pain, only superciliary corrugator EMG was analysed, because it represents a negatively valenced emotional expression. Electrodes of 4 mm ø Ag-AgCl (EL254S, Biopac Systems, Inc.) were used with a contact gel (GEL100, Biopac Systems, Inc.). Electrodes were connected to EMG100C amplifiers (Biopac Systems, Inc.) with the following acquisition settings: gain 500, low-pass filter 500 Hz, notch filter off and high-pass filter 10 Hz. Sampling was at 1000 Hz. The signal was further filtered in the Acqknowledge software using a band pass filter of 30–300 Hz to remove signal not owing to muscle activity. A band stop filter at 49–51 Hz was used to filter out line noise. Average-rectified EMG signal was determined. Recordings were downsampled to 100 Hz in order to decrease file size, and data were exported as text files. Before analyses, recordings were further downsampled to 10 Hz, using a LOESS curve in R. Responses were averaged over a time window of 2 s (figure 10*a*–*d*) and log-transformed before statistical analysis, in order to better approximate a normal distribution.

#### 2.3.8. Heart rate

We recorded heart rate in wave 2 only. A three-lead ECG was acquired by placing disposable Ag/AgCl electrodes (EL503, Biopac Systems, Inc.) on the right side of the neck, on the left upper arm and on the left ankle (ground reference). ECG100 amplifiers (Biopac Systems, Inc.) were used with the following settings: Gain 2000, mode R wave, 35 Hz LPN on, high-pass filter 0.5 Hz. Sampling was at 1000 Hz. Recordings were downsampled to 100 Hz in order to decrease file size, and data were exported from the Acqknowledge software as text files. Of the 39 participants from whom ECG was recorded, one was excluded owing to electrode disattachment and two were excluded owing to frequent extrasystoles. Heart rate was derived from raw curves by a peak finding algorithm in R. Estimated heart rate of less than 40 or more than 200 beats per minutes was rejected (0.2% of data). For each event, heart rate was normalized to the 2 s preceding stimulus onset and averaged over a time window from 2.5 to 4 s from stimulus onset.

### 2.4. Rating scales

#### 2.4.1. Interpersonal reactivity index

The interpersonal reactivity index (IRI) has four subscales which measure different dimensions of trait empathy: empathic concern (EC), perspective taking (PT), personal distress (PD) and fantasy (FS) [19,20]. The IRI has been validated in a Swedish context [50], although the four-factor structure could not be replicated. Instead, EC formed one factor and PT, PD and FS together formed another factor. For this reason, we have not analysed differences between IRI subscales. Two participants were excluded on this measure because they had a large and non-random number of missing items (owing to failing to turn over the page). One additional item response was missing, and it was imputed based on the mean of the subscale.

#### 2.4.2. Toronto alexithymia scale-20 (TAS-20)

The TAS-20 measures alexithymia, a construct thought to represent difficulties in identifying and describing one’s own emotions. It has three subscales: difficulty identifying feelings, difficulty describing feelings and externally oriented thinking [51]. We analysed only total scores. The scale has been validated in Swedish [52]. Four participants were excluded on this measure for failing to respond to a large number of the items. One additional item response was missing, and it was imputed based on the mean of the subscale.

#### 2.4.3. State-trait anxiety inventory

The state-trait anxiety inventory (STAI) has a state and a trait subscale [53]. We used a non-validated Swedish translation with which we have considerable experience, and which can be found in [54]. The state subscale (S) was administered before the experiment, and then again at the end of the experiment. For the trait subscale (T), four participants each missed one item. These data were imputed using the average of the remaining items, rounded to the nearest integer. For the state subscale, two participants were not administered the scale the second time. One participant gave three illegible responses and three participants each missed one item. Imputation was performed using the average of the remaining items.

#### 2.4.4. Psychopathy personality inventory-revised

The psychopathy personality inventory-revised (PPI-R) assesses psychopathic traits [55,56]. It contains eight content scales, which have been organized into a two-factor structure, encompassing the factors fearless dominance (FD; reflecting social poise, fearlessness and stress immunity) and self-centred impulsivity (SCI; reflecting impulsivity, irresponsibility and egocentricity). It also contains a subscale particularly reflecting lack of empathy (coldheartedness, C), which typically does not load highly on either factor. The Swedish version of the PPI-R has been validated based partly on the data collected in this study [57]. Missing responses were imputed based on the mean for each subscale [58] in 11 participants (7.5%). Three participants had high scores on the inconsistent responding subscale (greater than or equal to 45) and were excluded from analyses.

### 2.5. Analyses and data

Data and analysis code for this paper are openly available at [54]. In order to preserve anonymity, participants’ age and educational background have been omitted from the published dataset. All analyses were made with R [59], using the packages **RCurl** [60] to read data from GitHub, **quantmod** [61] to find ECG R wave peaks, **nlme** [62] to build mixed-effects models, **effects** [63] to obtain confidence intervals on estimates and **RColorBrewer** [64] for graphing. Mixed-effects models have been used throughout unless otherwise indicated. For reference, full output tables of regression models for main outcomes are also published at [54], for both waves together and for each separately. Results reported here have been previously made available as a preprint [65] and in a student thesis [66].

## 3. Results

### 3.1. Participants

Thirty-nine participants completed each wave. In addition, we tested eight participants as a pilot experiment before the main study began. Pilot participants are not included in any analyses. Their data are, however, published along with the other participants’ data (see Methods), as some measures may have value for reuse. For mimicry, in wave 1, two participants were excluded for technical reasons and one owing to facial tics, and in wave 2, one participant was excluded owing to facial tics. For empathy for pain, two participants were excluded from wave 1 after debriefing because it emerged that they had not understood the instructions, one participant was excluded owing to problems with the recording equipment, and a further three were excluded because they voiced suspicions about the nature of the confederate at debriefing. From wave 2, one participant was excluded, because he was found to have a psychiatric diagnosis after the experiment, four were excluded owing to not reaching VAS 80, and four were excluded because they voiced suspicions about the nature of the confederate at debriefing. Thus, the final number of participants included in either experiment was 76.

Participant characteristics are shown in table 1. In wave 1, the oxazepam group had higher ratings on the IRI-EC. Because the rating scale was completed approximately 20 min after drug administration, we had to consider the possibility that ratings were affected by the drug. To exclude this putative explanation, we asked the participants to complete the IRI again by mail after the experiment. 24 out of 35 participants responded (69%), and the mean change in IRI-EC was −0.02 (s.d. 0.51). Furthermore, in wave 2, we administered the IRI before drug administration, and then again with items in a scrambled order after drug administration, and found no difference in IRI-EC ratings owing to oxazepam (−0.04 [−0.28, 0.19], *p*=0.70). Thus, we conclude that the group difference in IRI-EC ratings in wave 1 was more likely owing to chance than to a drug effect. Main analyses in the empathy for pain experiment were performed with IRI-EC as a covariate in order to attempt to control for this imbalance between groups.

**Table 1.** Characteristics of participants. Means and standard deviations are given, unless otherwise indicated. Data refer to participants included in either of the two experiments. In parentheses on the *n* row are the numbers included for emotional mimicry and for empathy for pain, respectively. See Material and methods section for abbreviations of rating scales.

	wave 1		wave 2	
	placebo	oxazepam	placebo	oxazepam
*n*	18 (16, 17)	19 (19, 19)	17 (13, 16)	22 (20, 22)
age (median, range)	20 (18–28)	21 (18–27)	22 (18–44)	23.5 (18–41)
any tertiary education (*n*, %)	13 (72%)	16 (84%)	11 (65%)	18 (82%)
IRI-EC	3.21 (0.67)	3.78 (0.48)	3.86 (0.60)	3.82 (0.32)
IRI-PT	3.29 (0.80)	3.67 (0.47)	3.62 (0.36)	3.45 (0.46)
IRI-PD	2.60 (0.58)	2.61 (0.70)	2.55 (0.45)	2.32 (0.56)
IRI-PT	2.96 (0.49)	3.33 (0.49)	3.33 (0.69)	3.27 (0.66)
STAI-T	40.7 (8.1)	38.2 (7.4)	39.3 (5.5)	35.2 (6.0)
TAS-20	45.3 (10.2)	40.9 (10.1)	40.4 (9.5)	37.1 (7.8)
PPI-R-SCI	140.2 (15.5)	148.1 (17.6)	158.2 (20.4)	140.4 (23.8)
PPI-R-FD	116.2 (22.1)	129.4 (20.3)	125.4 (10.7)	129.3 (14.5)
PPI-R-C	38.8 (5)	33.7 (4.1)	34.9 (4.4)	36.3 (5.1)

### 3.2. Efficacy of intervention

#### 3.2.1. Reaction times

**Figure 3.**
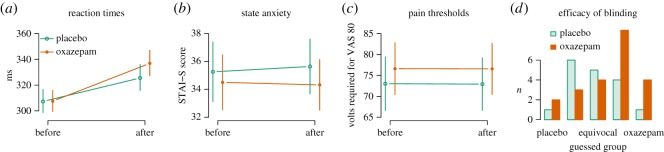
Efficacy of intervention. (*a*) Reaction times increased from before the experiment to after, and more so in the oxazepam group, confirming that the administered drug had a biological effect. Estimates were back-transformed from the inverse for plotting. (*b*) Oxazepam caused decrease state anxiety after the experiment in the oxazepam group compared with the placebo group. (*c*) Oxazepam did not affect participants’ pain thresholds. (*d*) Participants in wave 2 guessed after the experiment which treatment group they were in, using a five-level Likert-type scale to indicate whether they were sure they were in the placebo group, probably in the placebo group, equivocal, probably in the oxazepam group or sure they were in the oxazepam group. Labels are omitted for the ‘probably placebo’ and ‘probably oxazepam’ responses.

Oxazepam caused slower reaction times, seen as an interaction between treatment and first/second administration of the test (9.4 ms, [5.0, 13.8], estimates back-transformed from the inverse, *p*=0.0001, figure 3*a*), confirming biological activity of the drug. Reaction times were slower in the second test (25.0 ms, [22.3, 27.7], *p*<0.0001, figure 3*a*).

#### 3.2.2. State anxiety

Oxazepam caused decreased state anxiety, seen as an interaction between treatment group and first/second test (2.82, [−0.10, 5.73], *p*=0.03 (one-sided), figure 3*b*), further confirming expected drug activity. No change in anxiety from the first to the second test time was seen (−0.91, [−2.89, 1.06], *p*=0.36), nor any main effect of oxazepam (−2.06, [−7.10, 2.98], *p*=0.42).

#### 3.2.3. Pain thresholds

Oxazepam did not cause increased pain thresholds, seen as an interaction between treatment group and first/second test (−0.31 *V*, [−4.34, 3.72], *p*=0.88, figure 3*c*), confirming the expected lack of analgesic effect. No change in pain thresholds from first to second test time was seen (−0.21 *V*, [−3.03, 2.62], *p*=0.88) nor any main effect of oxazepam (−3.28, [−13.92, 7.36], *p*=0.54).

#### 3.2.4. Efficacy of blinding

Participants were not able to guess significantly better than chance whether they had received oxazepam or placebo (1.0, [−0.0004, ∞], *p*=0.05, one-sided Wilcoxon rank sum test, figure 3*d*), although the effect was in the direction of detection of true group membership.

### 3.3. Emotional mimicry

#### 3.3.1. Facial muscle activity

**Figure 4.**
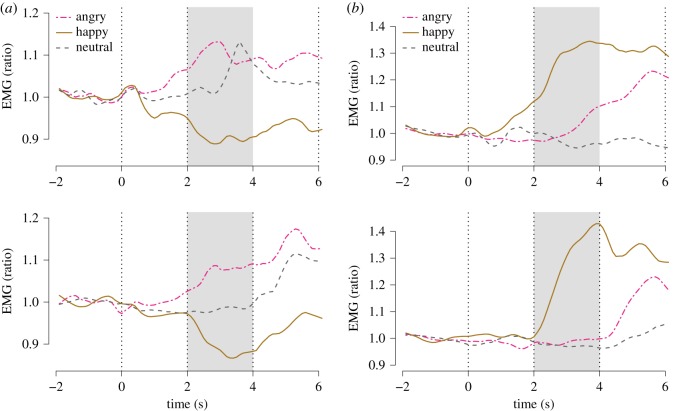
Emotional mimicry: EMG time-courses. (*a*) Corrugator. (*b*) Zygomatic. Top: wave 1. Bottom: wave 2. First vertical line: onset of video clip. Second vertical line: onset of emotional expression. Third vertical line: end of video clip. Shaded box: time window for effect averaging (2–4 s). Every response was indexed to mean activity in the 2 s preceding video clip onset (−2 to −0 s).

**Figure 5.**
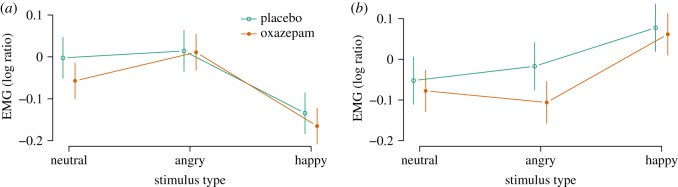
Emotional mimicry: effects of oxazepam. (*a*) Corrugator responses. (*b*) Zygomatic responses.

**Figure 6.**
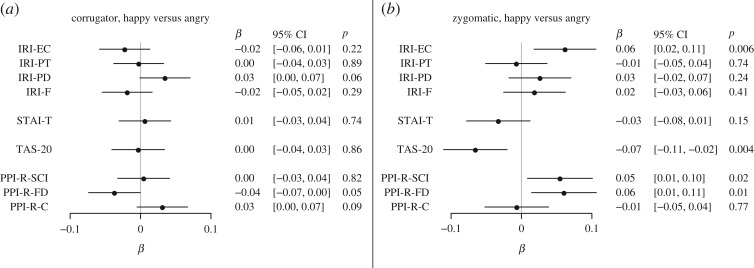
Emotional mimicry: personality predictors. (*a*) Corrugator responses. (*b*) Zygomatic responses. Effects shown are standardized regression coefficients with 95% CIs. Effects were investigated as interactions between rating scales and happy versus angry stimuli, and consequently, negative values mean more mimicry for corrugator responses and less mimicry for zygomatic responses.

EMG activity was analysed in the time window 2–4 s after stimulus onset as a ratio to the average activity during the 2 s before stimulus onset (figure 4). Happy stimuli caused decreased corrugator responses (−0.14 [−0.19, −0.09], *p*<0.0001, figure 5) and increased zygomatic responses (0.14 [0.07, 0.20], *p*<0.0001, figure 6), as expected. Angry stimuli did not cause significantly increased corrugator responses (0.02 [−0.04, 0.07], *p*=0.56, figure 5) nor decreased zygomatic responses (0.03 [−0.03, 0.09], *p*=0.33, figure 5). Following Dimberg *et al.* [67], we analysed the interaction of treatment with the effect of happy versus angry faces as the measure of mimicry, and found no significant effects for corrugator (−0.03 [−0.10, 0.04], *p*=0.44, figure 5) nor zygomatic (0.07 [−0.01, 0.16], *p*=0.10, figure 5) responses. In these analyses, oxazepam inhibited zygomatic responses across happy and angry conditions (−0.09 [−0.18, −0.00], *p*=0.04), but did not have a significant main effect on corrugator responses (−0.01 [−0.08, 0.06], *p*=0.83).

#### 3.3.2. Predictors of mimicry

Rating scales for personality measures were investigated as predictors of mimicry by analysing the interaction of the z-transformed score on each respective scale with the effect of happy versus angry faces, following Dimberg [67]. The fearless dominance subscale of the PPI-R predicted less corrugator mimicry (figure 6). The fearless dominance and self-centred impulsivity subscales of the PPI-R predicted more zygomatic mimicry and the Toronto alexithymia scale-20 predicted less zygomatic mimicry (figure 6).

### 3.4. Empathy for pain

#### 3.4.1. Rated unpleasantness

**Figure 7.**
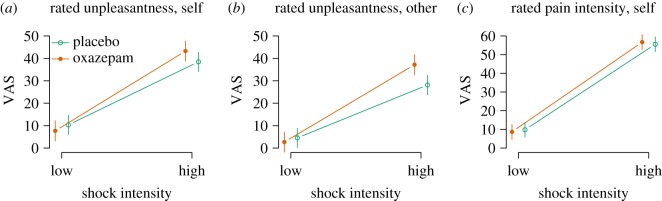
(*a*–*c*) Empathy for pain: Rated experience.

Shocks to other were rated less unpleasant than shocks to self (−5.8, [−7.9, −3.7], *p*<0.0001, figure 7*a*,*b*). Shocks of high intensity were rated more unpleasant than shocks of low intensity (28.1, [26.0, 30.3], *p*<0.0001, figure 7*a*,*b*). There was no main effect of oxazepam on rated unpleasantness (−2.6, [−9.0, 3.7], *p*=0.41). Shock intensity and self/other condition interacted such that high-intensity stimuli were rated less unpleasant in the other condition (−4.6, [−7.7, −1.4], *p*=0.004, and oxazepam interacted with high stimulus condition, with ratings increased by 7.4 [4.3, 10.5], *p*<0.0001, figure 7*a*,*b*).

The effect of oxazepam on empathic responding was assessed as a three-way interaction between treatment, shock intensity and self/other condition. We had hypothesized that oxazepam would cause lower-rated unpleasantness specifically in the other high condition, but this effect was not seen (3.5, [−0.9, 7.9], *p*=0.12, figure 7*a*,*b*).

A post hoc test in the self condition only showed a main effect of high pain stimulus of 28.1 [26.1, 30.2], *p*<0.0001, a main effect of oxazepam of −2.6 [−10.4, 5.3], *p*=0.52, and an interaction of 7.4 [4.4, 10.4], *p*<0.0001, with higher ratings in the high condition in the oxazepam group. A post hoc test in the other condition only showed a main effect of high pain stimulus of 19.9 [18.1, 21.7], *p*<0.0001, a main effect of oxazepam of −3.0 [−8.9, 2.9], *p*=0.31 and an interaction of 13.1 [10.5, 15.7], *p*<0.0001, with higher ratings in the high condition in the oxazepam group.

#### 3.4.2. Rated intensity

Rated pain intensity was not affected by oxazepam (−1.1, [−6.9, 4.6], *p*=0.70, figure 7*c*). As expected, rated pain intensity was higher to high shock intensity (46.8, [44.0, 47.5], *p*<0.0001, figure 7*c*). Oxazepam did not interact with shock intensity (2.3, [−0.2, 4.8], *p*=0.07, figure 7*c*).

#### 3.4.3. Skin conductance

**Figure 8.**
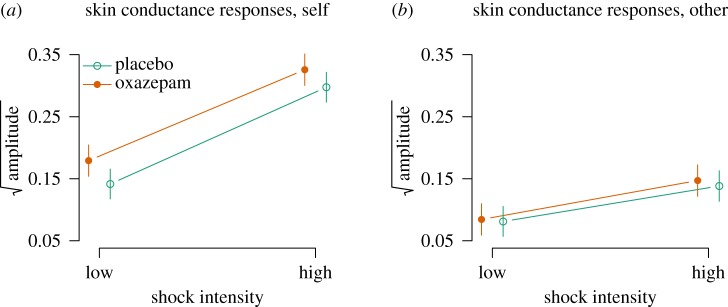
(*a*,*b*) Empathy for pain: skin conductance responses.

There were main effects of other versus self condition (−0.06, [−0.08, −0.05], *p*<0.0001) and of high versus low shock intensity (0.16, [0.14, 0.17], *p*<0.0001), and a two-way interaction (−0.10, [−0.12, −0.08], *p*<0.0001, figure 8*a*,*b*), such that skin conductance responses were highest in response to high-intensity shocks and to self. Oxazepam had a main effect on skin conductance (0.04, [0.02, 0.07], *p*=0.04, and oxazepam interacted with other versus self condition, with an effect of other condition of −0.03 [−0.05, −0.01], *p*=0.02, figure 8*a*,*b*).

The effect of oxazepam on empathic responding was assessed as a three-way interaction between treatment, shock intensity and self/other condition. We had hypothesized that oxazepam would cause lower skin conductance responses specifically in the other high condition, but this effect was not seen (0.02, [−0.02, 0.05], *p*=0.35, figure 8*a*,*b*).

A post hoc test in the self condition only showed a main effect of high pain stimulus of 0.16 [0.14, 0.17], *p*<0.0001, a main effect of oxazepam of 0.04 [−0.01, 0.08], *p*=0.08 and an interaction of −0.01 [−0.03, 0.01], *p*=0.38, with lower effect in the high condition in the oxazepam group. A post hoc test in the other condition only showed a main effect of high pain stimulus of 0.05 [0.03, 0.06], *p*<0.0001, a main effect of oxazepam of 0.00 [−0.03, 0.03], *p*=0.92 and an interaction of 0.01 [−0.01, 0.03], *p*=0.36, with lower effect in the high condition in the oxazepam group.

#### 3.4.4. Heart rate

**Figure 9.**
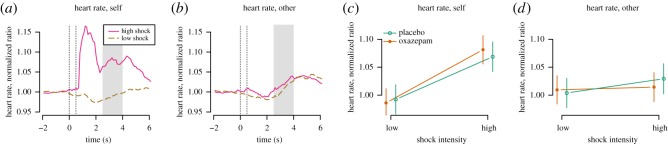
Empathy for pain: heart rate. (*a*,*b*) The first dotted vertical line shows the onset of the stimulus cue. The second dotted vertical line shows the onset of the shock and the shock cue. The grey area shows the time window for which signal was averaged. (*c*,*d*) In the self high condition, there was a large peristimulus peak. We did not include this peak in the time window for further analysis, because it may represent non-cardiac signal sources such as the electrical pain stimulus itself and associated muscle activity.

There was a main effect of high versus low shock intensity (0.076, [0.049, 0.104], *p*<0.0001), but not of other versus self condition (0.012, [−0.016, 0.039], *p*=0.41) and a two-way interaction (−0.051, [−0.091, −0.011], *p*=0.01, figure 9*a*,*b*), such that heart rate responses were highest in response to high-intensity shocks and to self. Oxazepam did not have a main effect on heart rate (−0.006, [−0.044, 0.032], *p*=0.74, figure 9*a*,*b*).

The effect of oxazepam on empathic responding was assessed as a three-way interaction between treatment, shock intensity and self/other condition. We had hypothesized that oxazepam would cause lower heart rate responses specifically in the other high condition, but this effect was not demonstrated (−0.039, [−0.093, 0.014], *p*=0.15, figure 9*a*,*b*).

A post hoc test in the self condition only showed a main effect of high pain stimulus of 0.08 [0.05, 0.10], *p*<0.0001, a main effect of oxazepam of −0.01 [−0.05, 0.03], *p*=0.76 and an interaction of 0.02 [−0.02, 0.06], *p*=0.31, with higher effect in the high condition in the oxazepam group. A post hoc test in the other condition only showed a main effect of high pain stimulus of 0.02 [−0.00, 0.05], *p*=0.10, a main effect of oxazepam of 0.01 [−0.03, 0.04], *p*=0.74 and an interaction of −0.02 [−0.06, 0.02], *p*=0.30, with lower effect in the high condition in the oxazepam group.

#### 3.4.5. Superciliary corrugator activity

**Figure 10.**
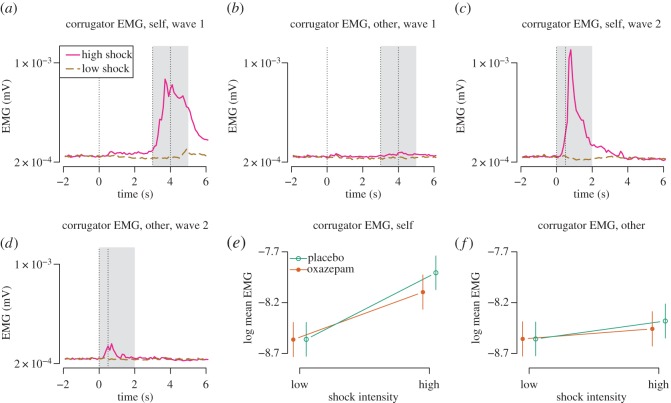
Empathy for pain: corrugator EMG activity. Because stimulus timing differed between waves 1 and 2, different time windows were used. (*a*,*b*) The first dotted vertical line shows onset of the stimulus cue. The second and third dotted vertical line bound the interval in which the shock and the shock cue appeared. The grey area shows the time window for which signal was averaged. (*c*,*d*) The first dotted vertical line shows the onset of the stimulus cue. The second vertical line shows when the shock and the shock cue appeared. The grey area shows the time window for which signal was averaged. (*e*,*f*) Estimates from mixed-effect models.

There was a main effect of high versus low shock intensity (0.66, [−0.39, −0.29], *p*<0.0001) but not of other versus self condition (0.00, [0.26, 0.36], *p*=0.91), and a two-way interaction (−0.48, [−0.58, −0.38], *p*<0.0001, figure 10*a*,*b*), such that corrugator EMG responses were highest in response to high-intensity shocks and to self. Oxazepam did not have a main effect on EMG responses (−0.00, [−0.25, 0.24], *p*=0.98, figure 10*a*,*b*), but it did show a two-way interaction with shock intensity (−0.19, [−0.28, −0.09], *p*=0.0001, figure 10*a*,*b*), such that responses to shocks of high intensity were lower in the oxazepam group.

The effect of oxazepam on empathic responding was assessed as a three-way interaction between treatment, shock intensity and self/other condition. We had hypothesized that oxazepam would cause lower corrugator EMG responses specifically in the other high condition, but this effect was not seen (0.11, [−0.02, 0.22], *p*=0.11, figure 10*c*,*d*).

A post hoc test in the self condition only showed a main effect of high pain stimulus of 0.66 [0.58, 0.73], *p*<0.0001, a main effect of oxazepam of 0.02 [−0.25, 0.29], *p*=0.88, and an interaction of −0.19 [−0.30, −0.08], *p*=0.0008, with lower effect in the high condition in the oxazepam group. A post hoc test in the other condition only showed a main effect of high pain stimulus of 0.12 [0.08, 0.16], *p*<0.0001, a main effect of oxazepam of −0.04 [−0.29, 0.21], *p*=0.76 and an interaction of −0.04 [−0.19, 0.34], *p*=0.12, with lower effect in the high condition in the oxazepam group.

#### 3.4.6. Predictors of empathic responding

**Figure 11.**
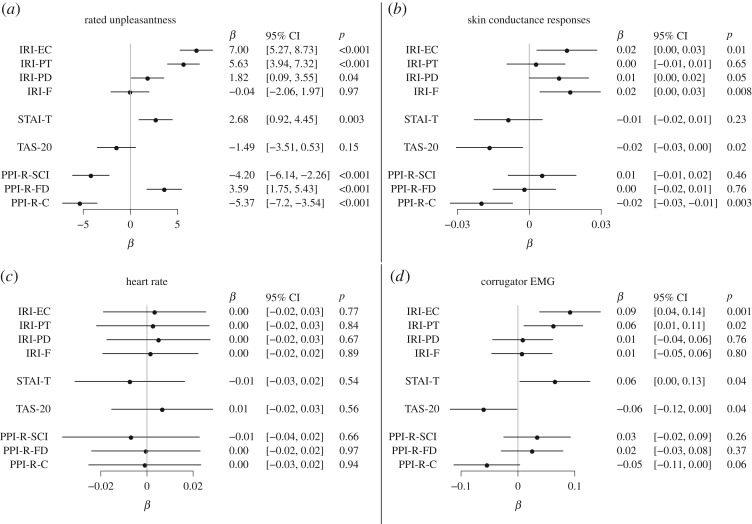
(*a*–*d*) Empathy for pain: personality predictors. Associations between PPI subscales and empathic responding have been reported previously [57].

We hypothesized that IRI-EC would predict empathic responses. Associations between PPI-R and empathic responding have been reported previously [57]. Predictors for responding in the empathy condition (high-intensity stimulus to the other person) are shown in figure 11. IRI subscales predicted increased empathic responding on ratings, skin conductance and EMG, but not heart rate. Conversely, TAS-20 predicted lesser empathic responses on ratings, skin conductance and EMG, but not heart rate. Besides the rating scales, we also investigated rated likability of the confederate, and it did not predict empathic responses on unpleasantness (2.24 [−0.16, 4.64], *p*=0.07), skin conductance responses (0.017, [−0.020, 0.013], *p*=0.11), corrugator EMG (−0.065, [−0.134, 0.004], *p*=0.07) nor heart rate (0.003, [−0.017, 0.023], *p*=0.80).

### 3.5. Adverse events

The shock electrode caused minor dermal injuries measuring up to approx. 1 mm at greatest diameter to 11 participants. Use of this electrode has been discontinued. Of the 39 participants from whom we recorded ECG, two were found to have irregular heart rhythm and were recommended to consult a physician.

## 4. Discussion

Oxazepam showed expected effects on reaction times and self-rated anxiety, confirming biological activity of the drug. The experimental paradigms showed expected main effects, confirming their validity. While subjective ratings may have been affected by demand characteristics, i.e. participants rating in a manner they believe to be expected of them, physiological measures were probably not much affected by such biases, because the participants were not well aware of the nature of the recordings.

### 4.1. Emotional mimicry

Oxazepam inhibited zygomatic EMG responses in response to both angry and happy stimuli. This finding implicates GABA as a regulatory neurotransmitter for facial emotional expressions, but the present results do not allow conclusions as to whether this regulation is specific or whether decreased responses occurred as a consequence of generally reduced vigilance. As in [67], mimicry was defined as the difference in activity between happy and angry stimuli. Thus, while oxazepam inhibited zygomatic EMG responses across stimulus categories, it did not inhibit mimicry of the major zygomatic muscle.

Diazepam (15 mg) has been previously found to impair identification of angry and fearful faces [38,39]. Similarly, another investigation found global impairment by 15 mg diazepam of identification of emotional faces [40]. In two other studies, 2 mg lorazepam [41] and 5 mg diazepam [42] did not effect recognition of facial emotional expressions, consistent with a dose-dependent effect. The effect of benzodiazepines on emotional mimicry has not, to the best of our knowledge, been investigated before.

Individual propensity for emotional mimicry has been previously shown to correlate to personality measures. Self-rated empathy using the questionnaire measure of emotional empathy (QMEE), the balanced emotional empathy scale (BEES), and the IRI has been found to predict emotional mimicry to emotional stimuli measured by EMG [67–72], as well as by scoring of videotaped expressions [73]. No effect was found in one experiment using a questionnaire on feelings after watching a video of a woman ill with AIDS [74]. Kurzius & Borkenau [75] found that Big 5 traits were related to emotional mimicry as judged by observers. Sonnby-Borgström [76] found, somewhat surprisingly, that alexithymia predicted greater mimicry. Conversely, Hermans *et al.* [77] found greater mimicry in participants with low autism quotient scores, though the effect was restricted to female participants.

### 4.2. Empathy for pain

Oxazepam did not inhibit empathic responses to others’ pain. Oxazepam did cause increased ratings of unpleasantness across stimulus conditions. This would seem to be at odds with the anxiolytic effects for which oxazepam is used. One explanation could be that oxazepam caused increased sleepiness, which is known to cause worse ratings of subjective experience [78]. While oxazepam is not mainly prescribed for its hypnotic properties, our reaction time results showed that participants in the oxazepam group did show a decrease in psychomotor vigilance, consistent with this interpretation.

The present results are similar to the finding by Olofsson *et al.* that 20 mg oxazepam did not influence event-related potentials in response to emotional images [79]. On the other hand, Siepmann *et al.* found that 0.5 mg lorazepam caused decreased skin conductance responses to aversive stimuli in humans, however, with no significant effects on pupil dilation, vigilance or mood [80]. With regard to subjective ratings, we have previously reported that 0.015 mg kg^−1^ midazolam decreased unpleasantness ratings to aversive pictures and the effect was reversed by 0.25 mg flumazenil [81].

Wang *et al.* [82] showed, using magnetic resonance spectroscopy, that higher levels of GABA in the anterior insula, a key region for empathy, predicted higher self-reported trait empathy on the IRI empathic concern and perspective taking subscales. This finding suggests the hypothesis that increased GABA signalling in the anterior insula would cause greater empathic responding, i.e. an effect in the opposite direction from what we hypothesized. Our results do not, however, provide support for a behavioural correlate of the finding by Wang *et al*.

We found that subscales of the IRI predicted empathic responding, supporting the notion that our experimental paradigm caused participants to experience sharing of the other person’s emotion. We also found that the TAS-20 total score predicted less empathic responding. Previous neuroimaging studies have shown that TAS-20 scores predict both higher [83] and lower [84] responses in anterior insula to viewing others in pain. Both these studies, however, found that TAS-20 predicted lower behavioural responses to other’s pain, as we have found here.

It has been suggested that benzodiazepines are associated with violent criminal acts [23–25]. Based on these case series reports, we hypothesized that increased aggressive behaviour may relate to benzodiazepine-induced attenuation of empathic processing. It has been shown that activity in key regions in the empathy response, such as the insula [13], may be suppressed by lorazepam during emotional processing [85]. Also, in the present dataset, we observed a general attenuation of unpleasantness ratings for emotional pictures. However, because we observed no effects of oxazepam on ratings and physiological responses associated with empathy, our results do not support our initial hypothesis, even though our oxazepam dose was not pharmacologically ineffective. It is possible that higher doses are needed in order to achieve an effect on empathy processing. Alternatively, the choice of benzodiazepine compound may be a key factor. Future research will be required to investigate these possible explanations.

### 4.3. Limitations

One limitation of this study is that we do not know whether the observed lack of effect extends to other benzodiazepines, such as flunitrazepam and chlordiazepoxide, which have been proposed to cause aggressive behaviour. Furthermore, the facilitating effect of benzodiazepines on aggression seems to be potentiated by alcohol in real life and in the laboratory [24,86], whereas we have studied the effect of oxazepam in isolation, and not attempted to induce aggressive behaviour. Also, we cannot say whether a higher dose of oxazepam would have inhibited empathic responding. Finally, the nature of the participant sample (all-male, largely university students) limits generalizability of results.

## 5. Conclusion

Our experiment showed that 25 mg oxazepam inhibited neither emotional mimicry nor empathic responding, although it did inhibit zygomatic EMG responses across conditions during mimicry and increase ratings of unpleasantness during empathy for pain. These findings show that GABA signalling affects emotional processing, but they do not show specific effects of GABA potentiation on emotional mimicry nor empathy for pain. The present results do not provide additional evidence for the hypothesis that benzodiazepines inhibit empathy.

## Data accessibility

Data and analysis code for this paper are openly available at http://dx.doi.org/10.5281/zenodo.60385 [54].
